# *In vitro* and intracellular activities of frog skin temporins against *Legionella pneumophila* and its eukaryotic hosts

**DOI:** 10.1038/s41598-020-60829-2

**Published:** 2020-03-04

**Authors:** Alexandre Crépin, Jean-François Jégou, Sonia André, Florine Ecale, Anastasia Croitoru, Anne Cantereau, Jean-Marc Berjeaud, Ali Ladram, Julien Verdon

**Affiliations:** 1 Laboratoire Ecologie & Biologie des Interactions, UMR CNRS 7267, Université de Poitiers, 1 Rue Georges Bonnet, TSA 51106, 86073 POITIERS, Cedex 9 France; 2 Laboratoire Inflammation, Tissus Epithéliaux et Cytokines, UPRES EA4331, Université de Poitiers, 1 Rue Georges Bonnet, TSA 51106, 86073 POITIERS, Cedex 9 France; 3 Sorbonne Université, CNRS, Institut de Biologie Paris-Seine, IBPS, BIOSIPE, F-75252 Paris, France; 4 Laboratoire d’Optique et Biosciences, INSERM U1182 - CNRS UMR7645, Ecole polytechnique, 91128 PALAISEAU, Cedex France; 5 Laboratoire Signalisation et Transports Ioniques Membranaires, Université de Poitiers, 1 Rue Georges Bonnet, TSA 51106, 86073 POITIERS, Cedex 9 France

**Keywords:** Microbiology techniques, Microscopy, Antimicrobials, Bacterial host response

## Abstract

Temporin-SHa (SHa) is a small cationic host defence peptide (HDP) produced in skin secretions of the Sahara frog *Pelophylax saharicus*. This peptide has a broad-spectrum activity, efficiently targeting bacteria, parasites and viruses. Noticeably, SHa has demonstrated an ability to kill *Leishmania infantum* parasites (amastigotes) within macrophages. Recently, an analog of SHa with an increased net positive charge, named [K^3^]SHa, has been designed to improve those activities. SHa and [K^3^]SHa were both shown to exhibit leishmanicidal activity mainly by permeabilization of cell membranes but could also induce apoptotis-like death. Temporins are usually poorly active against Gram-negative bacteria whereas many of these species are of public health interest. Among them, *Legionella pneumophila*, the etiological agent of Legionnaire’s disease, is of major concern. Indeed, this bacterium adopts an intracellular lifestyle and replicate inside alveolar macrophages likewise inside its numerous protozoan hosts. Despite several authors have studied the antimicrobial activity of many compounds on *L. pneumophila* released from host cells, nothing is known about activity on intracellular *L. pneumophila* within their hosts, and subsequently mechanisms of action that could be involved. Here, we showed for the first time that SHa and [K^3^]SHa were active towards several species of *Legionella*. Both peptides displayed bactericidal activity and caused a loss of the bacterial envelope integrity leading to a rapid drop in cell viability. Regarding amoebae and THP-1-derived macrophages, SHa was less toxic than [K^3^]SHa and exhibited low half maximal lethal concentrations (LC_50_). When used at non-toxic concentration (6.25 µM), SHa killed more than 90% *L. pneumophila* within amoebae and around 50% within macrophages. Using SHa labeled with the fluorescent dye Cy5, we showed an evenly diffusion within cells except in vacuoles. Moreover, SHa was able to enter the nucleus of amoebae and accumulate in the nucleolus. This subcellular localization seemed specific as macrophages nucleoli remained unlabeled. Finally, no modifications in the expression of cytokines and HDPs were recorded when macrophages were treated with 6.25 µM SHa. By combining all data, we showed that temporin-SHa decreases the intracellular *L. pneumophila* load within amoebae and macrophages without being toxic for eukaryotic cells. This peptide was also able to reach the nucleolus of amoebae but was not capable to penetrate inside vacuoles. These data are in favor of an indirect action of SHa towards intracellular *Legionella* and make this peptide a promising template for further developments.

## Introduction

Natural antimicrobial peptides (AMPs) and derivatives thereof are promising alternatives to conventional antibiotics. They exhibit broad-spectrum activity and often induce rapid permeabilization/depolarization of cell membranes, making them less susceptible to resistance. Anurans (frogs and toads) skin secretions are a rich natural source of AMPs^1^. These peptides, also known as host defence peptide (HDPs)^2,3^, have been extensively presented as promising candidates to struggle the antibiotic resistance crisis rising worldwide but also as good templates for developing new anticancer, antidiabetic and immunomodulatory agents^1,4,5^. Because short peptides are ideal candidates for therapeutic application in terms of manufacturing cost^6^, frog skin peptides such as temporins are particularly interesting^7^.

Temporins are short natural HDPs (8–17 amino acid residues)^1,8–11^ and represent an abundant family (more than 120 members listed in the Antimicrobial Peptide Database^12^) of low cationic hydrophobic and C-terminally amidated peptides produced by ranid frogs. Temporins are described to be mainly active against Gram-positive bacteria, including some antibiotic-resistant clinical isolates (e.g. methicillin-resistant staphylococci and antibiotic-resistant enterococci)^13^. However, the therapeutic potential of some temporins also extends to Gram-negative bacteria. This is notably the case of Temporin-Tl, isolated from the frog species *Rana temporaria*^14^ and, the tetradecapeptide temporin-DRa from *Rana draytonii*^15^. Two other temporins, temporin-SHa and temporin-SHd, found in the skin secretions of the North African ranid frog *Pelophylax saharicus*^10,16^, also target efficiently Gram-negative bacteria. However, with only 13 amino acid residues, temporin-SHa has a much broader spectrum, compared to temporin-SHd (17 amino acid residues), being potent against *E. coli* and *A. baumannii* (like temporin-SHd) but also against *S. enterica* serovar Enteritidis and *K. pneumoniae*^17^. Many temporin analogs containing both natural and unnatural amino acids were designed for structure-activity relationship studies. Some of them, including [P^4^, D-L^11^]temporin-Tl (containing a leucine^11^ D-enantiomer)^18^, [Aib^14^]temporin-DRa (an alpha-aminoisobutyric acid in position 13)^19^ and [*p*-^*t*^BuF^5^, R^7^]SHf (4-tert-butyl-phe^5^)^20^, are of particular interest because highly potent against Gram-negative species and non-toxic towards mammalian cells.

Only a few temporins are active against human parasites^10,16,17,21,22^ and viruses^23,24^. Temporins SHa and SHd were reported to have potent activity against the human protozoan parasite *Leishmania*^10,16,17^, the etiological agent of leishmaniasis, a vector-borne tropical disease leading to morbidity and mortality^25^. Their antiparasitic activity was assessed against several *Leishmania* species responsible for visceral or cutaneous leishmaniases (*L. infantum*, *L. major*, *L. tropica*, *L. amazonensis* and *L. braziliensis*), but also against other trypanosomatids that cause major diseases in humans: *Trypanosoma brucei gambiense* (sleeping sickness) and *T. cruzi* (Chagas disease)^10,17^. [K^3^]temporin-SHa, which was obtained by increasing the net positive charge of SHa from +2 to +3, has been described as a potent antiparasitic peptide active against all these trypanosomatids, including antimony-resistant *L. infantum* parasites^17^. This analog showed broad antimicrobial spectrum, targeting also several Gram-negative bacteria and *Candida* species^17^. Regarding viruses, although an activity against two pathogens of ectodermic animals was previously reported for temporin-Ta^26^, it is only recently that a virucidal activity against a human virus (i.e. herpes simplex virus type 1, HSV-1), was demonstrated for temporins Tb and SHa^23,24^. Significant reduction of the virus titer was obtained after preincubation of HSV-1 with these temporins.

Interestingly, temporins can target intracellular pathogens. However, only limited data about such activities are presently available and the mechanisms by which temporins exert their intracellular activity remain unknown. Temporins Tb and Ta were shown to kill both ATCC-derived and methicillin-resistant *S. aureus* (MRSA) clinical strains within human immortalized keratinocytes (HaCaT cells)^27^. They diffuse into keratinocytes without entering in the nucleus. Temporin-Tb was also shown to be active against HSV-1 replication in both HEK-293 (human embryonic kidney cells) and HeLa S3 (human cervix epithelial cells)^23^. Similar results were reported for temporins SHa and [K^3^]SHa in human primary keratinocytes, with no immunomodulatory effect on the cell antiviral response^24^. In addition to these antiviral properties on infected cells, temporins SHa and [K^3^]SHa were shown to kill *Leishmania* parasites (*L. infantum* amastigotes) within macrophages^17^, like temporin-SHd^10^. This leishmanicidal activity against intramacrophagic amastigotes was higher compared to the activity on extracellular forms (axenic amastigotes). This suggests either direct intracellular targeting of *Leishmania* amastigotes by temporins or other cell death mechanisms involving synergistic effects with intracellular messengers (like nitric oxide). In line with this, both peptides were demonstrated to trigger apoptotis-like death in *Leishmania infantum* in addition to their primary membranolytic mechanism^17^.

In this study, to provide additional data on the intracellular effects of temporins and to get further insight on the underlying mechanisms, we focused our attention on temporin-SHa and its analog [K^3^]SHa, using *Legionella pneumophila* as model of intracellular pathogen. *L. pneumophila* is an opportunistic Gram-negative human pathogen responsible for severe form of nosocomial and community-acquired pneumonia called Legionnaire’s disease^28^. *L. pneumophila* is ubiquitous in aquatic environments where it can persist within multispecies biofilms and phagotrophic protists like free-living amoebae^29,30^. Pathogen transmission to humans occurs after inhalation of contaminated aerosol particles that reach alveolar mucosa where *L. pneumophila* replicates in macrophages^28^. Among natural compounds described to be active against *Legionella* (biosurfactants, essential oils, proteins), several AMPs were reported to exhibit such an activity^31,32^. However, to the best of our knowledge, no studies regarding the activity of temporins against *Legionella* have been reported to date.

In this work, we determined the anti-*Legionella* properties of temporin-SHa and [K^3^]temporin-SHa towards different *Legionella* strains, including several *L. pneumophila* serogroups. The anti-*Legionella* mechanism was also evaluated. The cytotoxicity of temporins against the two main *L. pneumophila* eukaryotic hosts, human macrophages and free-living protozoa *Acanthamoeba castellanii*, was initially assessed, after which we analysed their effect on *L. pneumophila*-infected cells. Finally, we used Cy5-labeled temporin-SHa to discern by confocal fluorescence microscopy whether the peptide was located on the cell surface or into the infected cells, and to evaluate the peptide effect on intracellular *Legionella* multiplication. Immunomodulatory properties of temporin-SHa were also assessed by determining the mRNA expression levels of some pro-inflammatory cytokines and HDPs.

## Results

### SHa and [K^3^]SHa are potent anti-*Legionella* peptides

To assess the activity of SHa and [K^3^]SHa against bacteria that belong to the *Legionella* genus, several species of human health concern were grown in the presence of different amount of peptides and minimal inhibitory concentrations (MICs) were thus determined after a growth period of 96 h at 37 °C (Table 1). The SHa peptide was active against all the *Legionella* strains and exhibited low MIC values (3–6 µM). *L. feeleii*, *L. longbeachae* and *L. pneumophila* Paris were the more sensitive strains as the lowest concentration of SHa used was still able to inhibit their development (Table 1). Similar results were obtained for the [K^3^]SHa analog with MIC values in the same range as the parent peptide (Table 1). Taken together, these results indicated that both peptides have potent anti-*Legionella* activity and that SHa is slightly more effective than the [K^3^]SHa analog.

**Table 1:** Anti-*Legionella* activity of temporins SHa and [K^3^]SHa.

*Legionella* strains	MIC (µM)	
	SHa	[K^3^]SHa
*L. feeleii* ATCC 35072	≤1.56	3.12
*L. longbeachae* ATCC 33484	≤1.56	3.12
*L. pneumophila* Bloodmington-2 ATCC 33155 (Sg 3)	3.12	3.12
*L. pneumophila* HL 06041035 (Sg 1)*	3.12	6.25
*L. pneumophila* Lens CIP 108286 (Sg 1)	3.12	3.12
*L. pneumophila* Lorraine (Sg 1)*	3.12	3.12
*L. pneumophila* Los Angeles-1 ATCC 33156 (Sg 4)	3.12	6.25
*L. pneumophila* Paris (Sg 1)	≤1.56	3.12

Results are the mean of three independent experiments. Microbial strains were obtained from various culture collections: ATCC (American Type Culture Collection), CIP (Collection de l’Institut Pasteur de Paris, France) and *Centre National de Référence des Légionelles, Lyon, France. Sg: Serogroup.

**Figure 1:**
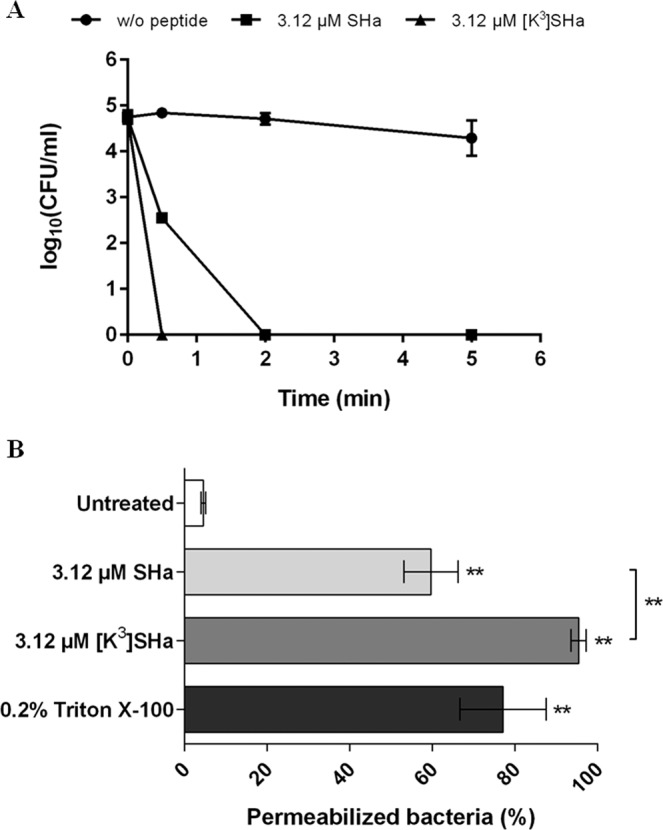
Bactericidal effect of SHa and [K^3^]SHa against *L. pneumophila* Lens (**A**) Time-kill curves of temporins. Exponential growth phase bacteria (10^5^ CFU/ml) were diluted in 10 mM sodium phosphate buffer (pH 6.9) and incubated 5 minutes with 3.12 µM SHa or [K^3^]SHa. Control corresponds to bacteria incubated in buffer without peptide. (**B**) Envelope permeabilization. Exponential growth phase bacteria (5.10^7^ CFU/ml) were diluted in 10 mM sodium phosphate buffer (pH 6.9) and incubated 5 minutes with peptides. Controls correspond to bacteria incubated in buffer without peptide (negative control) or with 0.2% Triton X-100 (positive control). Data were analyzed by nonparametric Mann-Whitney U test (two-tailed) using GraphPad Prism 6.0 software. **P < 0.01, compared to the untreated condition. Data represent the mean (±standard deviation, SD) of three independent experiments, each performed in duplicate. Error bars indicate SDs.

To determine whether temporin-SHa and [K^3^]SHa are able to kill *Legionella* sp. or only inhibit their growth, time-killing experiments were conducted using *L. pneumophila* Lens as a target. Indeed, this strain exhibits the same sensitivity to both temporins and is often used as a model when studying the effect of AMPs towards *Legionella* sp.^31^ At MIC concentration (3.12 µM), both peptides were shown to completely abolish the cultivability of *L. pneumophila* Lens (Fig. 1A). However, [K^3^]SHa analog was more effective than SHa because only 30 seconds were required to achieve complete loss of cultivability (2 minutes for SHa) (Fig. 1A). At this stage, even if we cannot conclude about the killing ability of temporins, our data indicated that temporins displayed a rapid bactericidal effect on *L. pneumophila*, most likely by permeabilizing bacterial cells. D-SHa (enantiomeric SHa) with all residues in the D-configuration was also synthesized and assayed towards *L. pneumophila* Lens. The MIC value determined after a growth period of 96 h at 37 °C was identical to that of the parent peptide (3.12 µM, data not shown) revealing intrinsic proteolytic stability of SHa in these incubation conditions and also that this peptide acts through a nonstereospecific (i.e. nonreceptor) mediated mechanism.

### SHa and [K^3^]SHa induce a rapid permeabilization of *L. pneumophila* cells

**Figure 2:**
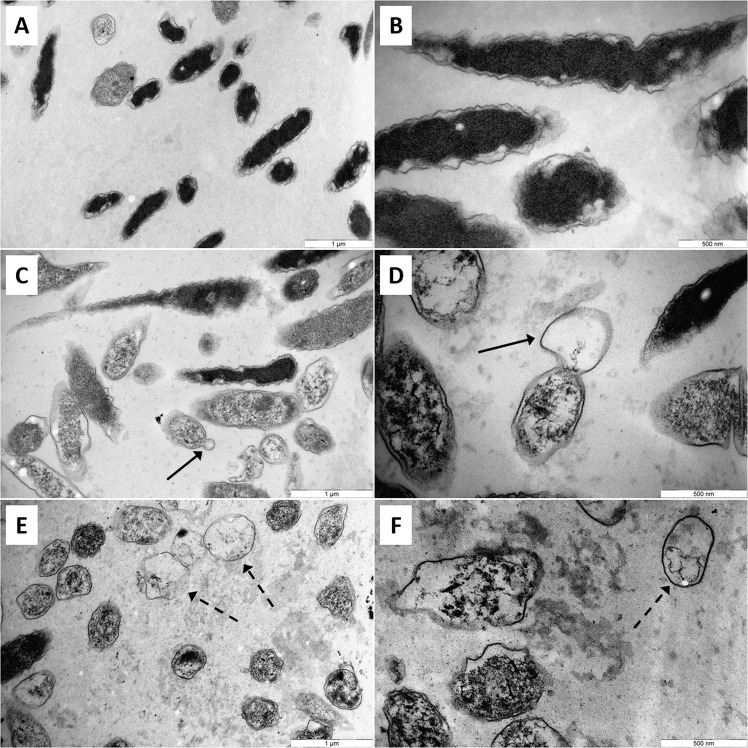
Transmission electron micrographs of *L. pneumophila* Lens exposed to temporins. Exponential growth phase bacteria (5.10^7^ CFU/ml) were diluted in 10 mM sodium phosphate buffer (pH 6.9) and incubated 5 minutes without peptide (**A,B**), with 3.12 µM SHa (**C,D**) or 3.12 µM [K^3^]SHa (E,F). Solid arrows indicate blebs on cell surface and dashed arrows indicate ghost-like structures.

The effect of temporins on bacterial cell integrity was further investigated by two complementary approaches. Firstly, we used propidium iodide to quantify the loss of bacterial membrane integrity in presence of 3.12 µM temporins. As indicated in Fig. 1B, both peptides were able to permeabilize the bacterial cell envelope. However, [K^3^]SHa analog displayed a more pronounced effect than the parent peptide. Indeed, 3.12 µM [K^3^]SHa were sufficient to permeabilize 95.4% (±2.1%) of *L. pneumophila* while at the same concentration, SHa induced only 59.7% (±7.3%) permeabilization. We then used a transmission electron microscopy (TEM)-based approach to gain insight into the effect of temporins on the morphology and the inner cell integrity of *L. pneumophila*. Microscopic observations of *L. pneumophila* control cells (untreated with temporins) showed well-formed bacilli of 1.5 to 2.0 μm long that contained numerous ribosomes in the cytoplasm (Fig. 2A,B). They exhibited a discernible envelope and some vacuoles were also visible. When bacteria were in contact with 3.12 µM SHa for 5 min, a loss of the intracellular organization was obtained as highlighted by a diminution of electron-dense regions (Fig. 2C). Many bacilli had lost their envelope integrity, and severe damages like membrane wrinkling and bubbling were also observed. These data indicated that SHa compromises the structural integrity of the cell wall, leading to cell lysis (Fig. 2C,D). Concerning [K^3^]SHa treatment, bacteria were also severely damaged by the peptide at a very low concentration (3.12 μM), as revealed by the presence of ghost-like structures containing large electron-lucent areas (Fig. 2E,F). Cells had lost a part of their cytoplasmic contents, although the overall cell shape was still recognizable. Altogether, our data are consistent with a high permeabilization potency of temporins towards *L. pneumophila* cells.

### SHa reduces the number of intracellular *L. pneumophila* in both amoebae and macrophages

The intracellular state is crucial for the natural multiplication process of *L. pneumophila*. We therefore investigated the capacity of SHa and [K^3^]SHa to efficiently kill intracellular forms of *L. pneumophila*. Prior to these experiments, the toxicity of both peptides towards *Acanthamoeba castellanii* and THP-1-derived macrophages cells was first determined (Supplemental Fig. S1). In our conditions, even if quite similar half maximal lethal concentrations (LC_50_) were observed for [K^3^]SHa (6.1 µM) and SHa (7.0 µM) against amoebae, SHa appeared to be less toxic against THP-1-derived macrophages as the determined LC_50_ was 12.3 µM (5.6 µM for [K^3^]SHa). Therefore, the following experiments were conducted only with this peptide. So, we examined if SHa was stable within the THP-1 culture medium. Using a LC-MS approach, we quantified the amount of SHa in RPMI medium just after dilution and also at the end of a 24 h incubation period at 37 °C. Hereafter, we assayed the activity of these samples against *L. pneumophila* Lens. LC/ESI-MS analysis of temporin-SHa in positive mode led to the observation of two intense pseudo-molecular ions at *m/z* 1380.6150 and 690.8583, which were assigned to the singly and doubly charged forms respectively (data not shown). We chose the pseudo-molecular ion at *m/z* 1380 to perform a calibration curve of SHa in serum-free RPMI medium before being able to quantify SHa after an incubation period at 37 °C. SHa was stable in this medium as a similar amount of peptide was present in our samples at t = 0 and t = 24 h (Supplemental Fig. S2A). Moreover, SHa was still active because an incubation of *L. pneumophila* Lens in these media samples for 1 h led to 80% decrease in cell viability (Supplemental Fig. S2B). Altogether, our results indicated that the slight loss of *L. pneumophila* load within THP-1-derived macrophages could be attributed directly or indirectly to SHa.

**Figure 3:**
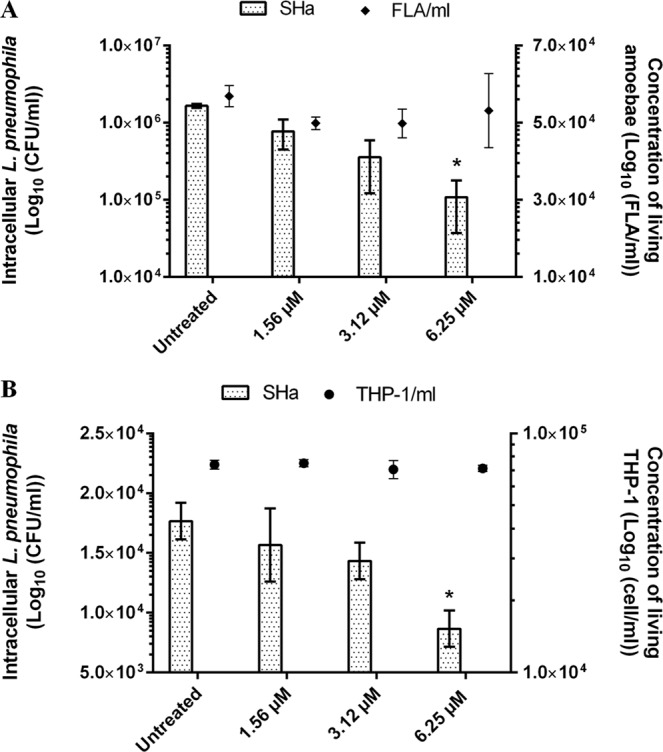
Survey of intracellular *L. pneumophila* Lens upon SHa addition. (**A**) *A. castellanii* were infected at MOI = 20 with GFP-expressing *L. pneumophila* Lens. FLA: free-living amoebae. (**B**) THP-1 cells were infected at MOI = 20 with GFP-expressing *L. pneumophila* Lens. Data were analyzed by nonparametric Kruskal-Wallis test followed by a Dunn’s multiple comparison *post-hoc* test using GraphPad Prism 6.0 software. *P < 0.05 for the 6.25 µM condition when compared to the untreated condition. Data represent the mean (±standard deviation, SD) of three independent experiments, each performed in triplicate. Error bars indicate SDs.

To address the question whether temporins could act on intracellular *L. pneumophila*, we followed the bacterial load inside cells upon SHa addition. *A. castellanii* and THP-1-derived macrophages were infected for 24 h with GFP-expressing *L. pneumophila* Lens (MOI = 20) and cells were simultaneously treated with SHa at different nontoxic concentrations, all being under LC_50_ values. SHa reduced the number of intracellular *L. pneumophila* within amoebae in a dose-dependent manner (Fig. 3A). At the highest concentration tested, more than 90% of *L. pneumophila* were killed. It is important to notice that the concentration of viable amoebae was only slightly affected by increased peptide concentrations (less than 5% mortality) and that the treatment did not compromise the viability of amoebae. In comparison, a lower bactericidal effect was obtained on intramacrophage *L. pneumophila* (52.3% of total population killed) at the highest SHa concentration tested (Fig. 3B). However, it should be stressed that *L. pneumophila* invasion induced a considerable reduction of the macrophage population (~90%) as the initial seeding concentration was 1.10^6^ cells per well, whereas the temporin treatment did not compromise the viability of remaining THP-1-derived macrophages (Fig. 3B).

### SHa penetrates inside both amoebae and macrophages, spread into cytoplasm but not into vacuoles

**Figure 4:**
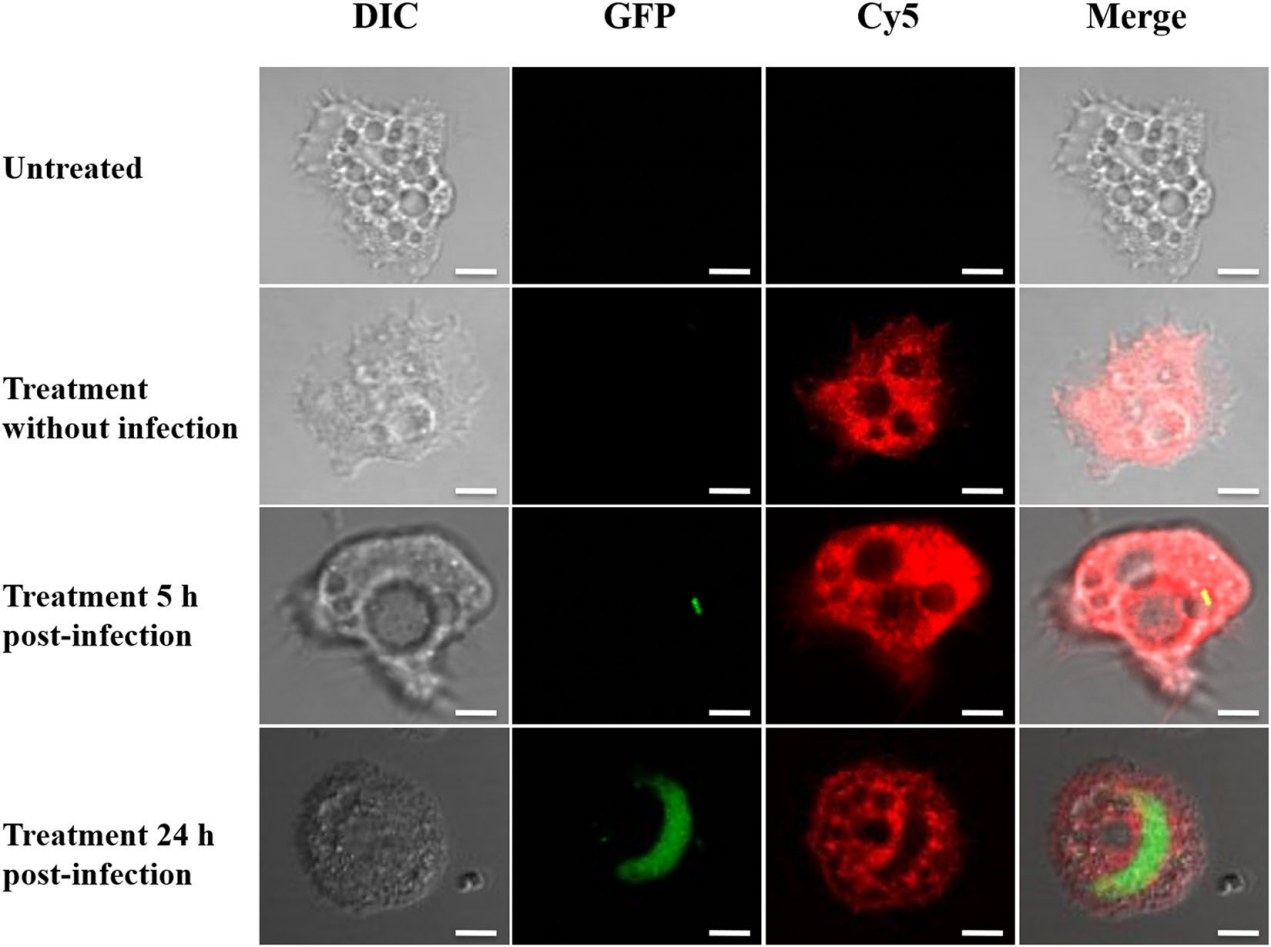
Confocal laser scanning microscopy images of infected *A. castellanii* cells (5 h or 24 h post-infection) with GFP-expressing *L. pneumophila* and treated with Cy5-SHa for 2 h. The first column shows differential interference contrast (DIC) images, the second one shows the GFP fluorescence signal from *L. pneumophila*, the third column shows the Cy5-SHa signal, and the last column shows the overlay of the two fluorescence signals. All bars represent 10 µm.

**Figure 5:**
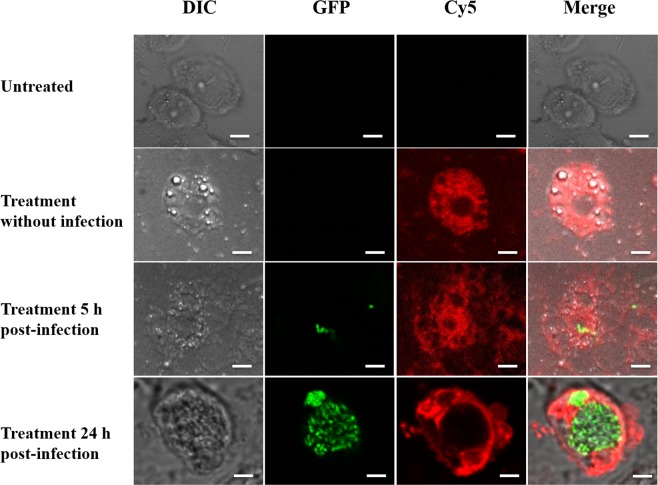
Confocal laser scanning microscopy images of infected THP-1-derived macrophages (5 h or 24 h post-infection) with GFP-expressing *L. pneumophila* and treated with Cy5-SHa for 2 h. The first column shows differential interference contrast (DIC) images, the second one shows the GFP fluorescence signal from *L. pneumophila*, the third column shows the Cy5-SHa signal, and the last column shows the overlay of the two fluorescence signals. All bars represent 10 µm.

**Figure 6:**
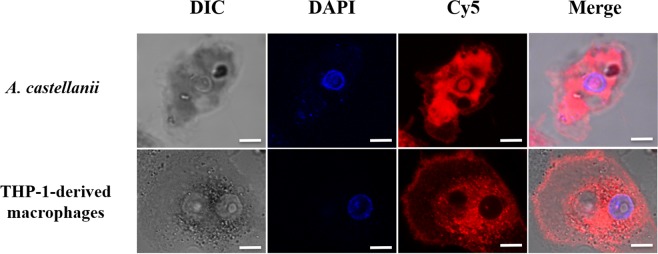
Confocal laser scanning microscopy images of *A. castellanii* and THP-1-derived macrophages treated with Cy5-SHa for 2 h. The first column shows differential interference contrast (DIC) images, the second column shows the DAPI fluorescence signal, the third column shows the Cy5-SHa signal, and the last column shows the overlay of the two fluorescence signals. All bars represent 10 µm.

To unravel whether temporin-SHa is able to enter into the cytoplasm of eukaryotic hosts used in this study and to be co-localized with *L. pneumophila*, a temporin-SHa labeled with the fluorescent dye Cy5 was used to conduct an imaging study by confocal fluorescence microscopy. We primary determined the MIC value of Cy5-SHa analog against *L. pneumophila* Lens to ensure that the C-terminal modification of the labeled analog had no impact on the biological activity. The mean of MIC values was found to be identical (3.12 µM) to that of the parent peptide (data not shown). The second point we focused on was the capacity of Cy5-SHa to interact with *L. pneumophila* host cells. The peptide was able to enter into the cytoplasm of *A. castellanii* within 12 min upon addition (Supplemental Fig. S3A and Supplemental Movie S1). Interestingly, the red signal was not evenly distributed as all vacuoles remained non-fluorescent (Fig. 4). Moreover, changes of shape occurred in treated non-infected cells as some cells became round. When amoebae were infected with *L. pneumophila*, bacteria were seen in vacuoles but again, the peptide red signal was only localized into cytoplasm. Amoebae had also adopted a characteristic round shape. Similar data were obtained with THP-1-derived macrophages inside which the peptide penetrated within 10 minutes and it was only localized outside vacuoles into the cytoplasm (Supplemental Fig. S3B and Fig. 5). In order to verify whether the internalization of the peptide is not due to the dye by itself, we have performed live imaging assays with both amoebae and THP-1-derived macrophages (Supplemental Fig. S3A,B). With each cellular model, a quick entry of Cy5 maleimide alone was recorded while it took few minutes to detect the red fluorescence when the Cy5-SHa was used. This time lag between the curves reflects the time necessary for SHa accumulation. Therefore, SHa is able to penetrate into the cytoplasm but not within vacuoles independently of whether intracellular *L. pneumophila* are present or not. Intriguingly, we observed that the cell nucleolus of amoebae was also strongly labeled by the red fluorescence within 20 to 30 minutes (Fig. 6). This subcellular localization of SHa seems specific as nucleoli of macrophages remained unstained even after an observation period of 2 hours.

### THP-1-derived macrophages do not produce antimicrobial peptides in response to SHa

To complete this analysis and further determine if the slight reduction of *L. pneumophila* load in macrophages could be due to additional factors, we have investigated the immune response of THP-1-derived macrophages by quantifying gene expression of proinflammatory markers (alarmin IL-1α, IL-8) and HDPs representative of different families of cationic peptides expressed by human macrophages (S100A7, also known as psoriasin, cathelicidin LL-37, human-β-defensins hBD-1 and hBD-3) (Supplemental Fig. S4). As expected, upon addition of bacteria, a strong upregulation of the two proinflammatory markers known to be released by pathogen-activated macrophages, i.e. the IL-8 chemokine and the IL-1α, was observed at the mRNA level. However, the expression of these two markers remained unchanged when macrophages were exposed to SHa alone, as compared to the untreated control (Supplemental Fig. S4). When macrophages were infected with *L. pneumophila* and treated 24 h with SHa, no synergistic induction of mRNA of IL-8 and IL-1α was detected. Regarding the expression of HDPs, no significant difference between all our conditions was found, indicating that there is no induction of these peptides (Supplemental Fig. S4).

### Intracellular *L. pneumophila* are protected from the action of SHa within amoebae

**Figure 7:**
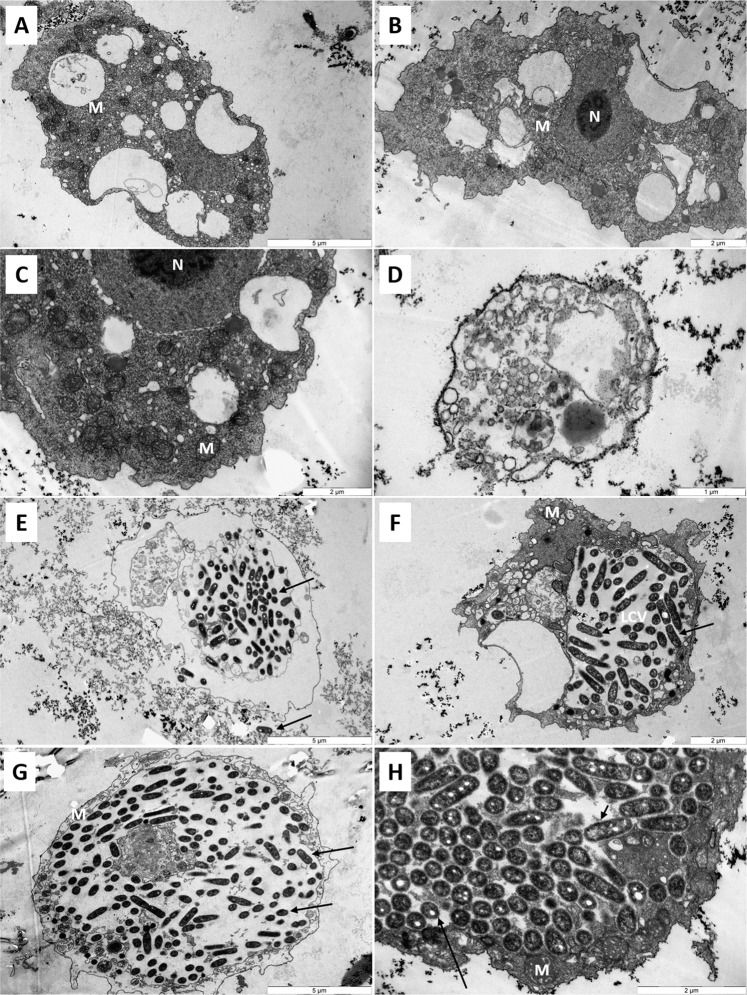
Transmission electron micrographs of *Acanthamoeba castellanii* infected or not with *L. pneumophila* Lens and treated or not with SHa. Untreated and uninfected cells (**A,B**), uninfected cells treated with 6.25 µM SHa (**C,D**), *L. pneumophila*-infected cells (**E,F**), *L. pneumophila*-infected cells treated with 6.25 µM SHa (**G,H**). LCV, *Legionella*-containing vacuole; M, mitochondria; N, nucleus. *L. pneumophila* are pointed with black arrows.

In order to notice whether the integrity of intracellular *L. pneumophila* could be compromise, observations using TEM were performed on infected amoebae because the highest loss of bacterial population was obtained with this model. Representative micrographs are gathered in Fig. 7. Microscopic observations of uninfected and untreated *A. castellanii* revealed large trophozoïtes (active stage) with well discernible membranes (Fig. 7A,B). Cells contained numerous vacuoles of variable sizes and harbored a cytoplasm filled with mitochondria. When cells were treated with 6.25 µM SHa, most of them were still well formed. No relevant morphological changes were observed (Fig. 7C). However, some amoebae were lysed as illustrated in Fig. 7D. The membrane integrity was lost as well as the intracellular organization. Amoebae appeared more electron-lucent as compared to untreated cells and many intracellular tiny vesicles can be seen. The addition of *L. pneumophila* resulted in bacteria entry into *A. castellanii*. Thus, *L. pneumophila* started to multiply within a unique cell compartment, the *Legionella*-containing vacuole (LCV) as illustrated in Fig. 7E,F. This enlarged vacuole was surrounded by numerous mitochondria. High *L. pneumophila* loads can be achieved before host death and then, bacteria could escape from this LCV. Vacuoles are visible inside bacteria and formed white patches. Finally, when *A. castellanii* were infected with *L. pneumophila* and then treated with 6.25 µM SHa, similar micrographs were obtained after observations of samples. Indeed, numerous dead or alive amoebae filled with bacteria were seen (Fig. 7G,H). Altogether, these data highlighted that *L. pneumophila* bacteria are protected from effects of SHa inside the LCV, indicating that the reduction of intracellular bacteria observed in this study is probably due to a host response rather than a direct effect of temporin.

## Discussion

HDPs from the temporin family are currently considered as good candidates for the generation of new class of peptide-based anti-infective therapeutics^1,8^. Biological activities of both parent peptides and analogs are widely described throughout the literature and include (i) direct broad-spectrum activity against bacterial, fungal or parasitic pathogens^9,10,16,17,21,22^, (ii) anti-Herpes simplex virus type 1 activity^23,24^, (iii) stimulation of human cells migration such as keratinocytes, monocytes and neutrophils^27,33^, (iv) endotoxin neutralization^34,35^, (v) chemoattraction of human phagocytes^33^; (vi) antibacterial/antiadhesive effect when grafted on surface^36,37^, (vii) antibiofilm properties^38,39^ and (viii) killing of intracellular microorganisms^10,17,27^. While temporins are mainly active against Gram-positive bacteria, only a few members exhibit a wider spectrum including Gram-negative bacteria, yeasts and parasites. Among those peptides, SHa^16^ has emerged as an interesting compound, with potent broad-spectrum activity, to study in depth the potency of temporins to kill intracellular organisms. Indeed, SHa and a recently designed analog with an increased positive net charge (from +2 to +3), [K^3^]SHa, were shown to efficiently kill intracellular forms of the human parasite *Leishmania*^17^. Moreover, they both inhibited intracellular steps of herpes simplex virus type 1 (HSV-1) replication in human primary keratinocytes^24^. Regarding activity of temporins against intracellular bacterial microorganisms, the only example concerns temporin-Tb, and also to a lesser extent temporin-Ta, that were described to be able to kill *S. aureus* within keratinocytes^27^. However, to the better of our knowledge, intracellular killing of Gram-negative bacteria was never demonstrated although several Gram-negative species are of clinical interest, i.e. some ESKAPE pathogens or *L. pneumophila*^40–42^. *L. pneumophila* is able to survive and to multiply inside several hosts, mostly aquatic protozoan, but accidentally inside human alveolar macrophages^43^. This capacity is fundamental for the *Legionella* pathogenesis and can lead to the development of Legionnaires’ disease, a severe and sometimes fatal pneumonia. *L. pneumophila* adopt a biphasic and reciprocal intracellular life cycle in which bacteria alternate between a replicative form and a distinct transmissive form^30^. The transmissive state is activated when nutrients become limiting within host cell and allowed bacteria to evade from this host, then survive in the extracellular environment until the invasion of a new appropriate host^30,44–46^. Moreover, *L. pneumophila* is armed with a Dot/Icm type IV secretion system with over 330 proteins secreted that represent over 10% of the bacterium proteome^47^. This astonishing number of secreted proteins is believed to enable *L. pneumophila* to better replicate inside numerous hosts. We therefore focused our interest on this intriguing Gram-negative bacterium in association with two overused models, *Acanthmoeba castellanii* and THP-1-derived macrophages, and wondered if temporins could target both extracellular and intracellular *L. pneumophila*.

First, the activity of both temporins was evaluated on extracellular *Legionella* sp. in order to determine inhibitory concentrations. SHa and [K^3^]SHa were able to totally inhibit the growth of *Legionella* sp. at similar concentrations (MICs mainly between 3.12–6.25 µM). Moreover, all *Legionella* tested were sensitive including different species of clinical importance, like *L. feeleii*^48^ and *L. longbeachae*^49^, but also different serogroups of the major clinical species, *L. pneumophila*. Up to now, only the susceptibility of six Gram-negative strains (*A. baumannii*, 2 strains of *E. coli*, *K. pneumoniae*, *P. aeruginosa* and *S. enterica*) was tested and published data showed that [K^3^]SHa was more efficient than the parent peptide^17^. Indeed, MICs values for SHa ranged from 6 µM (*A. baumannii* ATCC 19606) to 50 µM (*P. aeruginosa* ATCC 27853) while they were equal to 3 µM or 6 µM for [K^3^]SHa. *Legionella* sp. can thus be added to the short list of Gram-negative bacteria sensitive to temporins. In addition, MIC values determined in this study are in the same micromolar range as MIC values obtained for the most efficient AMPs already described in the literature as anti-*Legionella* agents, i.e. warnericin RK, δ-hemolysin I and II, and Phenol-Soluble Modulins (PSMs) α and β^50,51^. SHa and [K^3^]SHa can therefore be considered as potent anti-*Legionella* AMPs. Time-kill study revealed that both peptides displayed a fast-bactericidal effect on *L. pneumophila* Lens. The loss of *L. pneumophila* viability occurred within 30 s for [K^3^]SHa while 2 min were needed for SHa to achieve similar results. These data are in line with a previous study showing that the bactericidal activity of the [K^3^]SHa analog was more efficient^17^. To go further in studying the mechanism of action, we noticed that this rapid loss of viability was accompanied by a cell permeabilization, which could attain up to 95% for [K^3^]SHa at a concentration equal to the MIC. Visualization of this activity was provided by transmission electron microscopy. Indeed, *L. pneumophila* cells were deeply damaged by both temporins, and lost the structural integrity of their cell wall. Taken together, our data indicate that killing of *L. pneumophila* by SHa and its analog occurs through a membranolytic mechanism involving rapid permeabilization, as it was previously described^17^.

Before starting to study the intracellular activity of temporins, we determined whether SHa and [K^3^]SHa were toxic for eukaryotic host cells. At concentrations above 10 µM (3-fold above the MIC), both peptides were toxic for *A. castellanii* and, to a lesser extent, for THP-1 cells. Surprisingly, Raja and coworkers obtained different LC_50_ values towards human THP-1-derived macrophages for SHa (60 µM) and [K^3^]SHa (55 µM)^17^. This could be explained by the fact that we used a XTT-based colorimetric assay while they determined the cell viability using Trypan blue staining. Overall, a lower toxicity was determined for SHa in our conditions and so we decided to perform intracellular assays with this peptide only. SHa, when used at concentrations below LC_50_ values, can reduce the amount of *L. pneumophila* inside eukaryotic host cells in a dose dependent-manner. At the highest non-toxic dose tested (6.25 µM), more than 90% *L. pneumophila* were killed within amoebae and about 50% within macrophages. Similar results were obtained for temporin-Tb with *S. aureus*-infected HaCaT keratinocytes^27^. Di Grazia and collaborators recorded a bacterial decrease of 80% within 2 h at 16 µM, a dose 4 times higher than the MIC. However, as far as we know, it is the first time that such an intracellular activity was demonstrated for temporins acting on Gram-negative bacteria. It is also important to stress that we observed an early loss of macrophages during the infection with *L. pneumophila* even if remaining cells were not injured by SHa. Those data are fully in agreement with previous studies showing that protozoan hosts are much more tolerant than macrophages to *Legionella* infection, obviously because this bacterium grows rapidly in macrophages and can induces early apoptosis^44,52,53^. To distinguish whether SHa could be internalized into host cells or remained trapped at the cell surface, we used Cy5-SHa and visualized that the peptide was able to rapidly enter amoebae and macrophages. Cy5-SHa was able to evenly diffuse within cytoplasm but not inside vacuoles. Also, the peptide was shown to accumulate at cell nucleolus. The nucleolus, which is the largest subnuclear structure in eukaryotic nuclei, is the place of rRNA synthesis, assembly of ribosomes and diverse ribonucleoprotein particles^54^. Our data clearly indicate that Cy5-SHa can accumulate into the nucleus of living amoebae but not into the nucleus of living macrophages. Di Grazia and collaborators have previously shown that rhodamine-labeled temporins Tb and Ta were able to enter uninfected HaCaT keratinocytes within 30 min and to diffuse into the cytoplasm, but without entering into the nucleus^27^. What remains unclear is how SHa enters into nuclei, and why there is such a difference between amoebae and macrophages. To date, several proteins/peptides have been described to localize and accumulate in the nucleolus of living cells^54^. These molecules are cationic, highly basic and contain arginines and/or lysines. The presence of specific sequences enriched in those positively charged amino acids defines nucleolar localization signals (NoLSs)^55^. Remarkably, SHa does not have clusters of basic amino acids as it contains only one lysine and is C-terminally amidated. However, the accumulation of SHa inside nucleoli suggests that the peptide is able to locate its target inside the nucleus as no evidence of a barrier separating the nucleolus from the surrounding nucleoplasm were provided. It is proposed that this accumulation is due to high-affinity interactions with core nucleolar components^56^. Experiments are thus to be conducted to find the precise target of SHa inside nucleoli.

Although the mechanism of action responsible for the killing activity of intracellular *L. pneumophila* is not completely deciphered, we believe that it occurred through an indirect effect of SHa once internalized. As we cannot exclude side processes to be involved in this bacterial load decrease upon peptide treatment, we explored some cellular factors commonly involved in host/pathogen interaction using macrophages. A focus was therefore made on proinflammatory markers and additional HDPs that could be produced after *Legionella* addition and be present in the medium besides SHa. As expected, when macrophages were exposed to *L. pneumophila*, in the presence or the absence of SHa, they responded by an increased expression of IL-1α and IL-8. However, no induction of other HDP genes was detected, suggesting that SHa was the sole peptide present in the culture medium supporting the antibacterial activity in our *in vitro* infection conditions.

In summary, temporin SHa is a potent antibacterial peptide able to act on both extracellular and intracellular *L. pneumophila*. This frog HDP can kill internalized *L. pneumophila* within *A. castellanii* and THP-1-derived macrophages without being toxic for host cells. After addition to the medium, it rapidly penetrates cells and diffuses evenly in the cytoplasm but not inside vacuoles. Remarkably, it can also concentrate inside the nuclear compartment, more specifically in the nucleolus. Further studies will thus be necessary to better understand the mechanism of action subtending the killing of intracellular *Legionella* and the relationship between this killing and the absence of peptide in vacuoles where *L. pneumophila* replicates. Temporin-SHa and analogs thus represent interesting candidates to gain insight into eradication of pathogens inside living hosts.

## Methods

### Microorganisms, cell lines and culture conditions

Bacterial strains used in this study are listed in Table 1. *Legionella* strains were cultured at 37 °C either on buffered charcoal yeast extract (BCYE) agar plates for 96 h or in buffered yeast extract (BYE) liquid medium for 30 h under shaking (150 rpm). Growth of the strains was monitored by measuring the optical density of cultures at 600 nm. *Acanthamoeba castellanii* ATCC 30010 was obtained from the American Type Culture Collection. Axenic *Acanthamoebae* were grown in peptone yeast glucose (PYG) medium and incubated at 30 °C. THP-1 monocyte cells (ATCC TIB202) were grown in RPMI 1640 medium supplemented with 10% fetal calf serum (FCS), 100 u/mL penicillin, 100 µg/mL streptomycin (all from Thermo Fischer, Waltham, MA, USA) at 37 °C and 5% CO_2_. Cells were differentiated into macrophages with 50 ng/mL of phorbol 12-myristate 13-acetate (PMA) for 96 h. Treatment with temporins and infections with *L. pneumophila* were performed using cells cultivated in serum-free and antibiotics-free RPMI medium.

### Peptides synthesis and reagents

Temporin-SHa (SHa, FLSGIVGMLGKLF_NH2_), its enantiomer D-temporin-SHa (D-SHa, corresponding to SHa in the all-D α-C configuration), and [K^3^]temporin-SHa (SHa with Ser^3^ residue replaced with Lys), were synthesized using solid-phase standard Fmoc chemistry protocols, as previously described^57^ but with the following modifications. Synthesis was carried out on a CEM Liberty Blue automated microwave peptide synthesizer (CEM Corporation, Peptide Synthesis Platform, IBPS, Sorbonne University, Paris, France) using Protide Rink Amide LL resin (CEM Corporation, USA, 0.19 mmol/g substitution). Post-deprotection washing with N, N-dimethylformamide (DMF) was followed by coupling using a diisopropyl carbodiimide (DIC)/Oxyma activation method. The peptidyl resin was cleaved and deprotected by incubation (3 h at room temperature) with an acidic mixture containing 94% trifluoroacetic acid (TFA), 1% triisopropylsilane (TIS), 2.5% H_2_O and 2.5% 1,2-ethanedithiol (EDT). Resin was removed by filtration and the peptide was precipitated in cold ether. The crude material was then subjected to semi-preparative RP-HPLC on a Phenomenex Luna C18(2) LC column (100 Å, 250 × 10 mm). Elution was performed at a flow rate of 5 mL/min by a 20–70% linear gradient of ACN (0.07% TFA) in 0.1% TFA/water (1% ACN/min). Peptide purity was assessed by RP-HPLC using an analytical Aeris PEPTIDE column (XB-C18, 3.6 µm, 4.6 ×. 250 mm, Phenomenex). Elution was performed at 0.75 mL/min using the same linear gradient of acetonitrile mentioned above, followed by MALDI-TOF analysis (Mass Spectrometry and Proteomics Platform, IBPS, Sorbonne University, Paris, France). Fluorescent temporin-SHa (SHa-Cy5) was obtained from Genscript (Piscataway, USA) by synthesizing the analog [C^13^]temporin-SHa (FLSGIVGMLGKLC_amide_), and then by labeling thiol group of the Cys^13^ residue with maleimide-activated Cyanine5 dye. Control experiments were conducted with the Cyanine5 maleimide dye alone so it was purchased from abcam (Cambridge, United Kingdom).

### Antimicrobial assays

Activity against *Legionella* strains was assessed with a liquid growth inhibition assay performed in 96-well microtitration plates, according to a previously described protocol^50^. Minimal inhibitory concentration (MIC) was defined as the lowest concentration of temporins that fully inhibits the growth of a given *Legionella* strain after 96 h at 37 °C. MICs were determined as the mean value of three independent experiments, each performed in duplicate.

### Time-killing and envelope permeabilization assays

Time-killing and envelope permeabilization assays were conducted according to methods detailed elsewhere^58^. Temporins were used at a final concentration of 3.12 µM. Three experiments were carried out in duplicate for each assay.

### Intracellular killing of *L. pneumophila*

At confluence, *A. castellanii* were seeded in 24 wells plates at 5.10^4^ trophozoites/well one hour in Page’s Amoeba Saline solution (PAS). Once cells adhered, bacteria were added as follow. Cultures of GFP-expressing *L. pneumophila* Lens were picked from a plate, diluted in BYE medium (OD_600_ = 0.1) and grown overnight at 37 °C under constant shaking (180 rpm). Bacteria were washed and diluted in PAS buffer, and 100 μL of suspension was added to each well such that it contains 1 × 10^6^ bacteria (MOI = 20). Plates were incubated at 30 °C for 24 h. THP-1 cells were seeded into 24-well plates at a cell density of 1.0 × 10^6^ cells/well, and differentiated into macrophages using phorbol 12-myristate 13-acetate (PMA, 50 ng/ml, Sigma) for 72 h. Then cells were infected with bacteria to reach 2.10^7^ bacteria/well (MOI = 20) in RPMI medium without antibiotics at 37 °C for 24 h. Infections were synchronized by centrifugation at 500 × g, 30 °C for 10 min. The cells were washed 3 h post-infection to discard extracellular bacteria. Peptides, diluted in PAS buffer or RPMI, were added 5 h post-infection. 24 h post-infection cells were washed and resuspended in PBS by scraping for amoebae and by trypsin treatment during 10 min at 37 °C for macrophages. To break up, cells were vortexed with FastPrep-24™ 5 G, twice at 6 M/s during 30 s and 1 min in ice. Suspensions were then spread onto BCYE agar plates. After 72 h at 37 °C, the number of colony forming units (CFU) of *L. pneumophila* was counted. Each infection was carried out in triplicate.

### Transmission electron microscopy (TEM)

TEM analyses were conducted as previously described^58^.

### Confocal laser scanning microscopy

*A. castellanii* ATCC 30010 (5.0 × 10^4^ cells) or THP-1-derived macrophages (1.0 × 10^6^ cells) were infected with *L. pneumophila* Lens GFP at a MOI of 20 in a 8-well IbiTreat μ-slide microscopy chamber (Ibidi). When needed, a fresh DAPI staining solution was added at a final concentration of 9.3 nM. Images were acquired with CLSM from Olympus (FV1000). GFP and Cyanine 5 fluorescence were obtained sequentially with 488 nm and 543 nm laser line excitation and spectral detection between 500–530 nm and 555–655 nm respectively. Live imaging started directly after adding Cy5-SHa 5 or 24 h post-infection. Time lapse imaging was performed with planApo N x60 oil 1.4 NA objective and numerical zooming (800 × 800 pixels, 0.12 μm/pixel). One image was recorded every 15 seconds. Images and films were then analyzed using the ImageJ software.

### Cytotoxicity assay

PMA-induced THP-1 macrophages were previously treated with 10 µg/ml mitomycin C (Sigma-Aldrich, Saint-Louis, MO, USA) in RPMI for 2 h before treatment with SHa or [K^3^]SHa for 24 h. Cytotoxicity was measured using a XTT-based assay according to the manufacturer’s instructions (Roche). Amoebae were incubated with peptide for 24 h at 30 °C. After incubation, cells were washed twice with 1 ml of PAS and adherent cells were harvested and counted for each condition using FastRead counting slides 102 (Biosigma). LC_50_ (concentration of peptide inducing 50% cell death) was calculated with GraphPad Prism 6.0 software and corresponds to the mean obtained from three independent experiments carried out in triplicate.

### Transcriptional analysis

Transcriptional analyses by RT-qPCR were performed as described elsewhere^59^.

### Stability assay

Stability of temporin-SHa incubated in RPMI 1640 medium at 37 °C was assayed by LC/ESI-MS titration with a XevoQ-TOF (Waters, Milford, MA, USA) mass spectrometer as previously described^58^ but with minor modifications. A calibration curve was first built using serial dilutions of SHa in RPMI 1640 medium without fetal calf serum. A volume of 10 µl was injected for each sample. LC/ESI-MS mass spectra were performed in positive mode with a cone voltage ramping from 20 to 40 V. The spray voltage was set to 3.0 kV, the source temperature to 120 °C and the desolvation temperature to 450 °C. The LC separation was conducted on a ProSwift^®^ RP-1S (Dionex) analytical reverse- phase HPLC column (4.6 mm × 50 mm). Separation was carried out using a water/acetonitrile/formic acid 0.2% (v/v) solvent system. After an initial 2 min wash with 15% acetonitrile, elution was achieved at a flow rate of 0.6 ml/min with a 7 min linear gradient from 15 to 90% acetonitrile, followed by a 2 min wash with 90% acetonitrile. In a second step, 70 µg SHa were initially diluted in 1 ml RPMI and incubated 24 h at 37 °C. Samples were analyzed at t = 0 and t = 24 h. In parallel, exponentially growing *L. pneumophila* Lens were added to each sample (t = 0 and t = 24 h) at a final concentration of 5.10^7^ CFU/ml. Then, 70 µg SHa (50 µM) were added to each bacterial suspension. Samples were incubated for 45 min at 37 °C and stained with propidium iodide (PI) for 15 min in the dark at room temperature. Measurements were performed on a CytoFLEX flow cytometer (Beckman Coulter, Roissy, France). A total of 30,000 events were recorded in each sample. Three experiments were carried out and the patterns were reproducible.

## Supplementary information

Supplementary information.

Supplementary information2
