# Supplementary material for: In vitro and intracellular activities of frog skin temporins against Legionella pneumophila and its eukaryotic hosts
Source: Sci Rep. 2020 Mar 4;10:3978. doi: 10.1038/s41598-020-60829-2 (PMC7055270; doi:10.1038/s41598-020-60829-2)
Supplement: Supplementary file 1 — Supplementary information. [file 41598_2020_60829_MOESM1_ESM.docx]

***In vitro* and intracellular activities of frog skin temporins against *Legionella pneumophila* and its eukaryotic hosts**

**Alexandre Crépin**^1^**,** **Jean-François Jégou**^2^**,** **Sonia André**^3^**,** **Florine Ecale**^1^**,** **Anastasia Croitoru**^4^**,** **Anne Cantereau**^5^, **Jean-Marc Berjeaud**^1^**,** **Ali Ladram**^3^**, and** **Julien Verdon**^1*^

^1^Laboratoire Ecologie & Biologie des Interactions, UMR CNRS 7267, Université de Poitiers, 1 Rue Georges Bonnet, TSA 51106, 86073 POITIERS Cedex 9, France

^2^Laboratoire Inflammation, Tissus Epithéliaux et Cytokines, UPRES EA4331, Université de Poitiers, 1 Rue Georges Bonnet, TSA 51106, 86073 POITIERS Cedex 9, France

^3^Sorbonne Université, CNRS, Institut de Biologie Paris-Seine, IBPS, BIOSIPE, F-75252 Paris, France

^4^Laboratoire d’Optique et Biosciences, INSERM U1182 - CNRS UMR7645, Ecole polytechnique, 91128 PALAISEAU Cedex, France

^5^Laboratoire Signalisation et Transports Ioniques Membranaires, Université de Poitiers, 1 Rue Georges Bonnet, TSA 51106, 86073 POITIERS Cedex 9, France

^*^Corresponding author: [julien.verdon@univ-poitiers.fr](mailto:julien.verdon@univ-poitiers.fr)

**A**

**
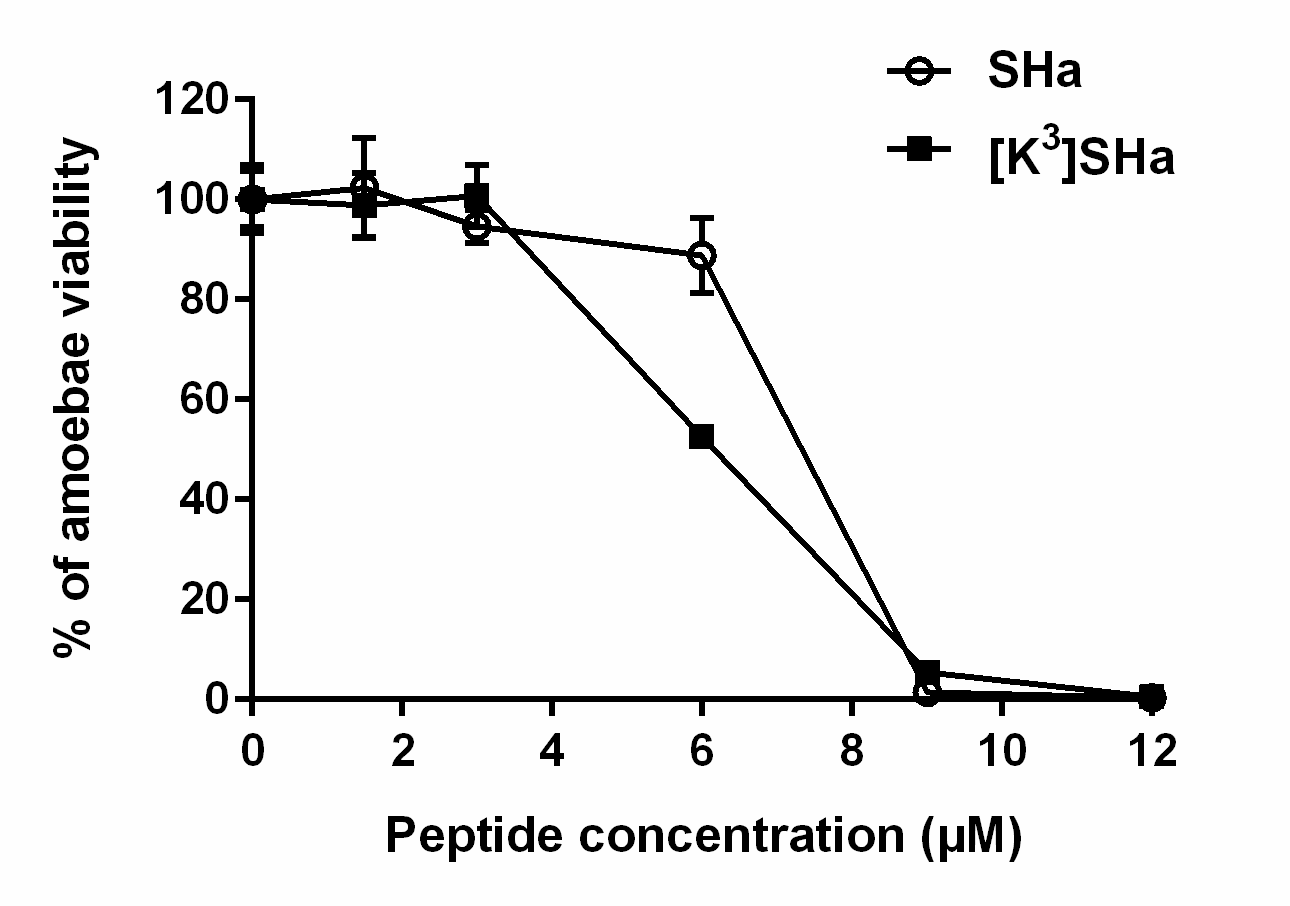
**

**B**

**
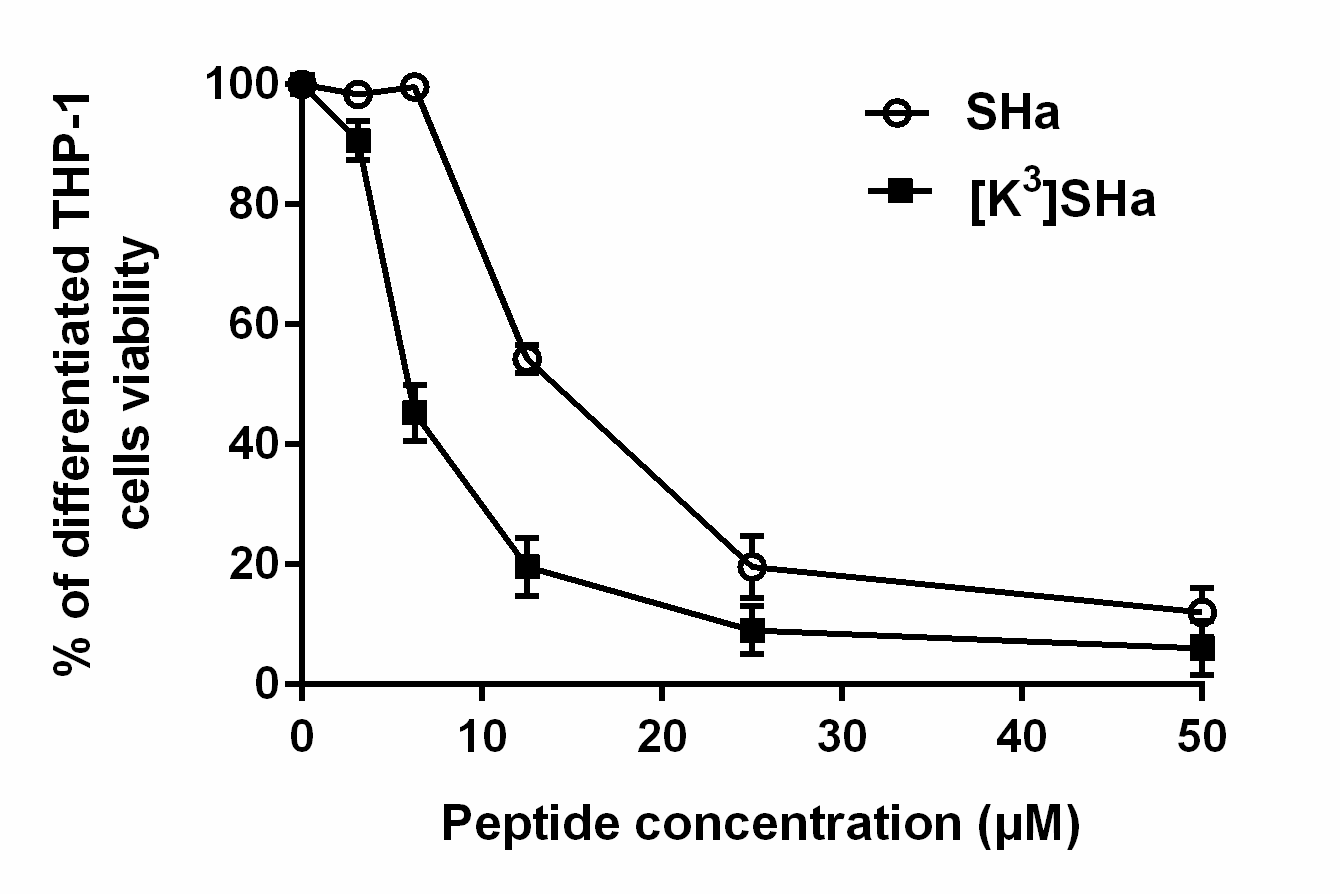
**

**Supplemental Figure S1.** Cytotoxic activity of temporins towards eukaryotic cells. (a) Percentage of free-living *Acanthamoeba castellanii* upon exposure to SHa or [K^3^]SHa as measured by adherent cell count, (b) Percentage of living THP-1-derived macrophages upon exposure to SHa or [K^3^]SHa as measured by the XTT assay. LC_50_ (half maximal lethal concentration) was determined with GraphPad Prism 6.0 software. Data represent the mean (± standard deviation, SD) of three independent experiments, each performed in triplicate. Error bars indicate SDs.

**A**


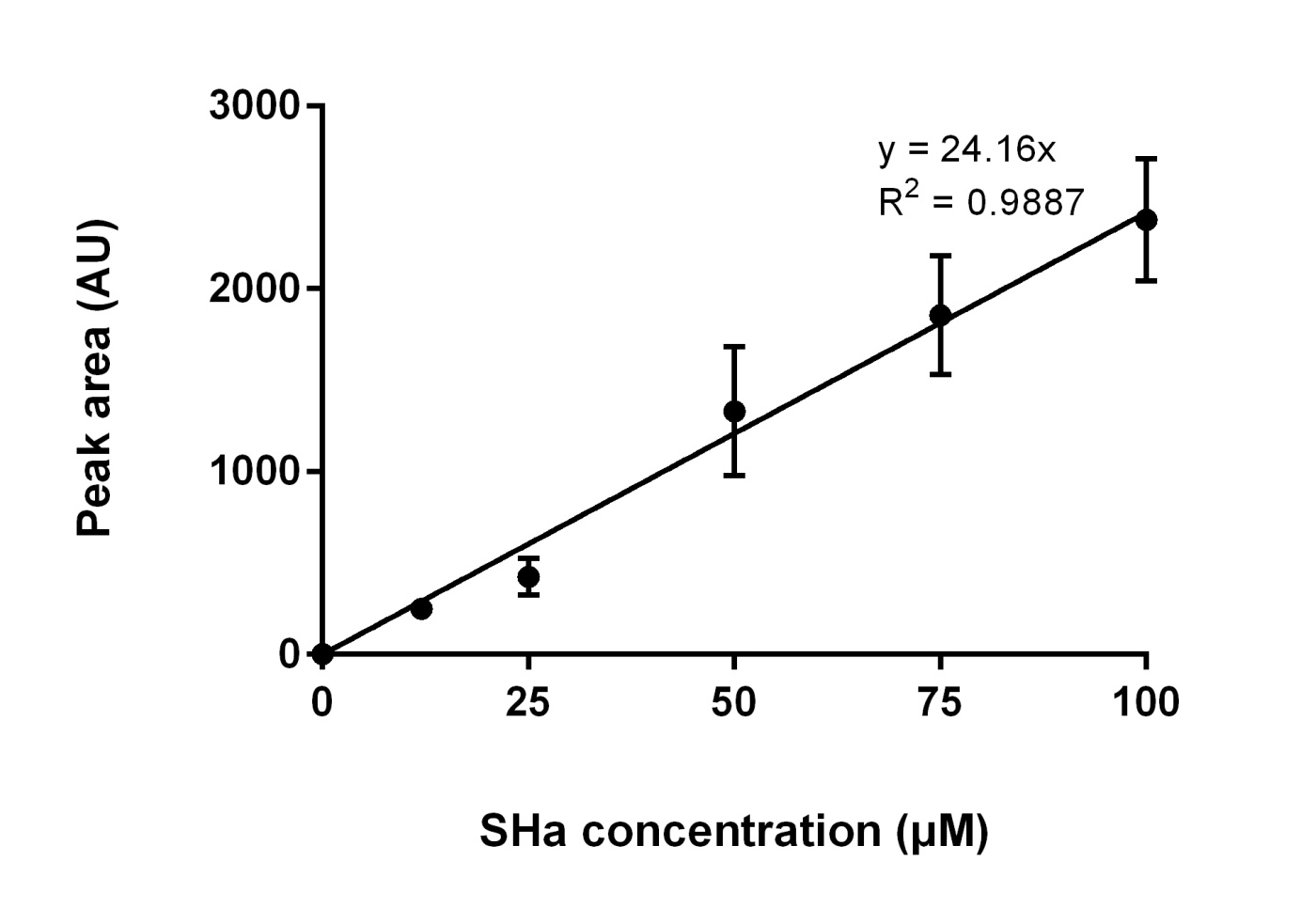


**B**

|  | **Mean of pic area (AU) (± SD)** | **Amount of peptide titred (µg) (± SD)** | **% of PI positive**  ***L. pneumophila*** |
| --- | --- | --- | --- |
| **T = 0 h** | 1682.08 (± 261.70) | 69.6 (± 10.8) | 4.6 (± 0.6) |
| **T = 24 h** | 1875.12 (± 222.74) | 77.6 (± 9.2) | 83.8(± 0.1) |

**Supplemental Figure S2.** Stability of temporin-SHa incubated in RPMI medium at 37°C. (A) Calibration curve of SHa in RPMI medium assayed by LC-MS and using the pseudo-molecular ion in positive mode [M+H]^+^ at *m/z* 1380. (B) LC/ESI-MS titration of SHa after 24 h incubating in RPMI medium using the pseudo-molecular ion in positive mode [M+H]^+^ at *m/z* 1380. Peptide control corresponds to 0 h incubation in RPMI. The percentage of propidium iodide (PI) positive *L. pneumophila* is also indicated following 1 h incubation with SHa previously incubated in RPMI (0 or 24 h at 37°C). Data represent the mean (± standard deviation, SD) of three independent experiments. Error bars indicate SDs.

**

**

**B**

**A**

**Supplemental Figure S3.** Fluorescence intensity of the Cy5 dye alone or the Cy5-SHa conjugate recorded by confocal laser scanning microscopy. (A) Uninfected *A. castellanii* cells (5.0 x 10^4^ cells) were exposed to 1 µM Cy5 maleimide or 6.25 µM Cy5-SHa. (B) Uninfected THP-1-derived macrophages (1.0 x 10^6^ cells) were exposed to 1 µM Cy5 maleimide or 6.25 µM Cy5-SHa. The Cy5 red fluorescence signal was recorded every 15 s and the percentage of intensity was calculated in relation to the maximum intensity measured. Data represent a representative experiment (from three independent experiments) performed in a single run.

**Supplemental Figure S4.** Expression profiles of cytokines and HDPs by THP-1-derived macrophages when exposed to either SHa, *L. pneumophila* (*Lp*) or both. Cells were collected after 24 h of treatment and analyzed for the expression of cytokines and antimicrobial peptides by RT-qPCR. M: THP-1-derived macrophages. Data are expressed as means ± SEM relative expression of the gene of interest to the housekeeping gene *GAPDH*, from three independent experiments. Data were analyzed by nonparametric Mann-Whitney U test (two-tailed) using GraphPad Prism 6.0 software. ** P < 0.01, compared to Macrophages alone.

**Supplemental Movie S1.** Confocal laser scanning microscopy live imaging of *A. castellanii* cells treated with 6.25 µM Cy5-SHa for 22 min. Images were taken every 15 s. Magnification x60.
